# Efficient biosynthesis of cinnamyl alcohol by engineered *Escherichia coli* overexpressing carboxylic acid reductase in a biphasic system

**DOI:** 10.1186/s12934-020-01419-9

**Published:** 2020-08-12

**Authors:** Chen Zhang, Qian Xu, Hongliang Hou, Jiawei Wu, Zhaojuan Zheng, Jia Ouyang

**Affiliations:** 1 Jiangsu Co-Innovation Center of Efficient Processing and Utilization of Forest Resources, College of Chemical Engineering, Nanjing Forestry University, Nanjing, 210037 People’s Republic of China; 2 Key Laboratory of Forestry Genetics & Biotechnology (Nanjing Forestry University), Ministry of Education, Nanjing, 210037 People’s Republic of China; 3 Jiangsu Province Key Laboratory of Green Biomass-based Fuels and Chemicals, Nanjing, 210037 People’s Republic of China

**Keywords:** Cinnamyl alcohol, Product inhibition, Biphasic system, Carboxylic acid reductase

## Abstract

**Background:**

Cinnamyl alcohol is not only a kind of flavoring agent and fragrance, but also a versatile chemical applied in the production of various compounds. At present, the preparation of cinnamyl alcohol depends on plant extraction and chemical synthesis, which have several drawbacks, including limited scalability, productivity and environmental impact. It is therefore necessary to develop an efficient, green and sustainable biosynthesis method.

**Results:**

Herein, we constructed a recombinant *Escherichia coli* BLCS coexpressing carboxylic acid reductase from *Nocardia iowensis* and phosphopantetheine transferase from *Bacillus subtilis*. The strain could convert cinnamic acid into cinnamyl alcohol without overexpressing alcohol dehydrogenase or aldo–keto reductase. Severe product inhibition was found to be the key limiting factor for cinnamyl alcohol biosynthesis. Thus, a biphasic system was proposed to overcome the inhibition of cinnamyl alcohol via in situ product removal. With the use of a dibutyl phthalate/water biphasic system, not only was product inhibition removed, but also the simultaneous separation and concentration of cinnamyl alcohol was achieved. Up to 17.4 mM cinnamic acid in the aqueous phase was totally reduced to cinnamyl alcohol with a yield of 88.2%, and the synthesized cinnamyl alcohol was concentrated to 37.4 mM in the organic phase. This process also demonstrated robust performance when it was integrated with the production of cinnamic acid from l-phenylalanine.

**Conclusion:**

We developed an efficient one-pot two-step biosynthesis system for cinnamyl alcohol production, which opens up possibilities for the practical biosynthesis of natural cinnamyl alcohol at an industrial scale.
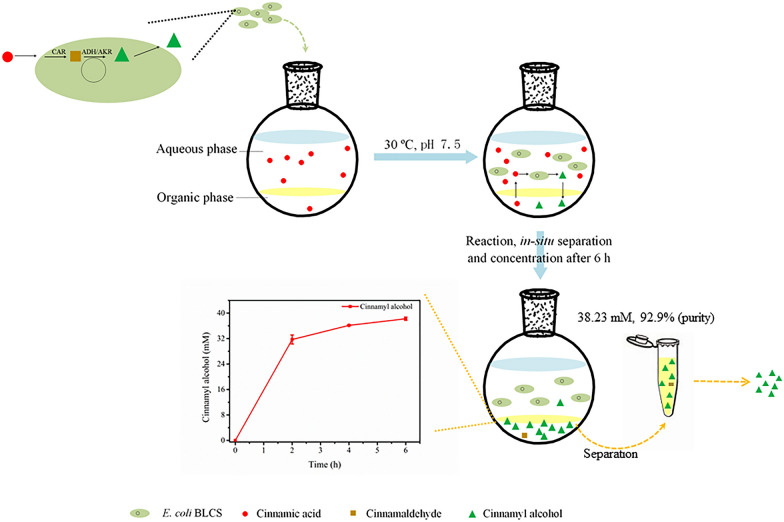

## Introduction

Recently, due to the rapid depletion of fossil fuels and the increasing demand for aromatic compounds, the production of these compounds from plant resources has increased in interest [1]. Monolignols, which are units of plant lignin, are important aromatic compounds that can be used to generate a wide range of high-value chemicals of commercial interest [2]. They have received much attention with the development of market requirements [3–6]. However, most natural monolignols are found in low concentrations in plants, and their extraction is limited by plant growth and expensive downstream processing costs. Industrial biosynthesis offers a promising alternative as it allows for scalable production of natural monolignols from bioresources [7].

Cinnamyl alcohol, a natural aromatic alcohol, is the skeleton of monolignols. Cinnamyl alcohol not only is used in the food and cosmetic industries due to its sweet-spicy odor and cinnamon taste, but also demonstrates good anti-inflammatory and antimicrobial activities [8–10]. In addition, cinnamyl alcohol is also a versatile chemical applied in the synthesis of various valuable compounds, such as cinnamyl esters (which are flavor and fragrance agents), flunarizine (which is used for fungal infection treatment), Taxol (which is a cancer treatment drug) and cinnamyl glycosides (which enhance immune function) [11–14]. Cinnamyl alcohol is also synthesized via the monolignol pathway in plants and the biotechnological production of natural cinnamyl alcohol has been reported by introducing the plant monolignol pathway into microbial strains [15]. As shown in Additional file 1: Figure S1, four enzymes involves in the synthesis of cinnamyl alcohol from l-phenylalanine in this pathway, including phenylalanine ammonia-lyase (PAL, EC 4.3.1.5), 4-coumarate: CoA ligase (4CL, EC 6.2.1.12), cinnamoyl-CoA reductase (CCR, EC 1.1.1.44) and cinnamyl alcohol dehydrogenase (CAD, EC 1.1.1.195) [16]. PAL catalyzes the conversion of l-phenylalanine to cinnamic acid, and 4CL catalyzes the subsequent conversion of cinnamic acid into CoA ester, which is reduced by CCR and CAD to cinnamyl alcohol. Zhou et al. constructed the pathway in *Escherichia coli* by overexpressing PAL, 4CL and CCR from different plant species. They found that endogenous alcohol dehydrogenases (ADHs) and aldo–keto reductases (AKRs) in *E. coli* could catalyze the reduction of cinnamaldehyde to cinnamyl alcohol, and the production of cinnamyl alcohol reached 2.13 mM [14]. Our group reported that a higher concentration of cinnamyl alcohol (4.8 mM) could be obtained using whole-cell biotransformation by introducing the pathway from *Populus trichocarpa* into *E. coli* BL21 (DE3) [17]. However, the current production of cinnamyl alcohol using this strategy is still unsatisfactory, as most characterized 4CLs demonstrated low activity towards cinnamic acid. Thus, a novel artificial approach mimicking the pathway of microbes (Additional file 1: Figure S1) was designed and a recombinant *Saccharomyces cerevisiae* strain coexpressing PAL2 from *Arabidopsis thaliana* (AtPAL2), carboxylic acid reductase from *Nocardia otitidiscaviarum* (NoCAR), and phosphopantetheine transferase from *E. coli* (EcSFP) was engineered for the bioconversion of l-phenylalanine to cinnamyl alcohol [2]. When endogenous reductases in *S. cerevisiae* were used, cinnamyl alcohol reached a maximum concentration of 0.83 mM when 1.35 mM cinnamic acid was added to the media. Klumbys et al. established a three-step cascade using PAL, CAR and ADH to synthesize approximately 4.3 mM cinnamyl alcohol from 5 mM l-phenylalanine after 27.5 h [18]. These findings suggested that biosynthesis of cinnamyl alcohol via the CAR pathway from microbes is potentially feasible and could avoid the problems associated with 4CL. However, reducing cinnamic acid to cinnamyl alcohol is still the limiting module in the cinnamyl alcohol biosynthesis pathway. To our knowledge, an enhanced biosynthesis strategy to overcome product inhibition has not been explored. There are no reports about the reduction of cinnamic acid to cinnamyl alcohol using heterologous CAR and endogenous ADHs or AKRs in *E. coli*.

Here, we report a new, high-yielding biphasic biotransformation for the one-pot two-step biosynthesis of cinnamyl alcohol from cinnamic acid. The recombinant *E. coli* strain BLCS, coexpressing *CAR* from *Nocardia iowensis* (*NiCAR*) and *SFP* from *Bacillus subtilis* (*BsSFP*), catalyzed the one-step reduction of cinnamic acid to cinnamic aldehyde. Cinnamaldehyde was then converted to cinnamyl alcohol by endogenous ADHs and AKRs. Subsequently, cinnamyl alcohol was indicated to cause severe product inhibition on its biosynthesis and a biphasic system was used to overcome this problem via in situ product removal for the first time.

## Materials and methods

### Materials

Isopropyl-β-d-1-thiogalactopyranoside (IPTG) and ampicillin were purchased from Sangon (Shanghai, China). Tryptone and yeast extract were purchased from Oxoid Co. Ltd. (Basingstoke, United Kingdom). Cinnamic acid and l-phenylalanine were purchased from Sigma-Aldrich (Shanghai, China). All other chemical reagents were of analytical grade and were commercially available.

### Plasmids, bacterial strains, and culture conditions

The plasmid and bacterial strains used in this study are listed in Additional file 1: Table S1. The *E. coli* BL21 (DE3) and recombinant strains used for gene cloning and biotransformation were cultivated in Luria–Bertani (LB) broth media supplemented with streptomycin or ampicillin at 200 rpm and 37 °C.

### Construction of recombinant strains

All DNA manipulation and general molecular biology techniques were conducted according to standard protocols. The *NiCAR* (GenBank number: Q6RKB1.1), *MpCAR* (CAR from *Mycobacterium phlei*, GenBank number: WP_003889896.1), *NoCAR* (GenBank number: WP_029928026.1) and *BsSFP* (GenBank number: WP_003234549.1) genes were codon-optimized and synthesized using a synthetic DNA service provider (General Biotechnology Co, Ltd., Chuzhou, China). Each *CAR* gene was inserted into the *Nco* I-*Bam*H I sites of a pCDFDuet-1 (multiple cloning site I, MCS I) vector to generate corresponding pCDFDuet-CARs. Thereafter, *BsSFP* was inserted into the *Bgl* II-*Kpn* I sites of MCS II of the pCDFDuet-CAR vector to generate pCDFDuet-NiCAR-BsSFP, pCDFDuet-NoCAR-BsSFP and pCDFDuet-MpCAR-BsSFP plasmids, which were subsequently introduced into *E. coli* BL21 (DE3), generating the corresponding recombinant strains *E. coli* BLCS, *E. coli* BLCS-N and *E. coli* BLCS-M.

### Biotransformation of cinnamic acid to cinnamyl alcohol by whole-cell catalysts in a monophasic or biphasic system

The recombinant *E. coli* cells were cultivated, washed and suspended in 100 mM sodium phosphate buffer (pH 7.5) for further study. The biotransformation reactions were performed in 50 mL flasks that contained 3 mL of a reaction mixture consisting of 100 mM sodium phosphate buffer (pH 7.5), *E. coli* cells, cinnamic acid and glucose. The comparison of the cinnamyl alcohol synthesis ability of different strains was performed under the following conditions for 4 h: 11 mM cinnamic acid, 55 mM glucose and different wet cells at an optical density of 50 (OD_600nm_ 50) in the reaction mixtures, 42 °C, and 200 rpm. To investigate substrate inhibition, reaction mixtures consisting of 5.7–18 mM cinnamic acid, 55 mM glucose and wet cells at an optical density of 50 (OD_600nm_ 50) were used. Biotransformation was performed at 42 °C with rotary shaking at 200 rpm for 2 h. To investigate production inhibition, reaction mixtures consisting of 8.5 mM cinnamic acid, 51 mM glucose, 0–5.5 mM cinnamic alcohol and wet cells at an optical density of 50 (OD_600nm_ 50) were prepared. Biotransformation was carried out at 30 °C with rotary shaking at 200 rpm for 2 h. The samples were heated to 100 °C and then centrifuged at 10,000*g* for 10 min. The concentrations of cinnamic acid, cinnamyl alcohol, cinnamaldehyde and 3-phenylpropanol in the supernatants were quantitatively analyzed by high-performance liquid chromatography (HPLC).

To explore the effects of organic solvent on the distribution coefficients, 3 mL of dibutyl phthalate, *n*-octane, *n*-hexane, *n*-octanol, ethyl acetate and *n*-hexanol was added to 3 mL of 100 mM PBS (pH 7.5) that included 18 mg of cinnamic acid, cinnamyl alcohol and cinnamaldehyde. The experiment was carried out at 30 °C with rotary shaking at 200 rpm, and samples were taken from different phases after 2 h.

The experiments to measure the effects of the phase ratio on the biosynthesis of cinnamyl alcohol were carried out as follows: wet cells at an optical density of 50 (OD_600nm_ 50) suspended in 3 mL of PBS (100 mM, pH 7.0) including 17.4 mM cinnamic acid (based on the aqueous phase), 102 mM glucose and wet cells at an optical density of 50 (OD_600__nm_ 50) and different volumes (0 mL, 0.6 mL, 1.2 mL, 1.8 mL, 2.4 mL, and 3 mL) of dibutyl phthalate were mixed together and incubated at 30 °C and 200 rpm for 6 h. The effects of substrate concentration (8.8–32 mM, based on the aqueous phase) were investigated on the basis of the optimized organic phase ratio. Samples were taken from different phases.

*V*_0_ (mM/h) was defined as the increase in concentration of cinnamyl alcohol during the initial reaction stage (1 h). The conversion and yield were calculated using the following equations:

$${\text{Conversion}}\left( \% \right) = \left( {{\text{Ns}}_{ 1} - {\text{Ns}}_{ 2} } \right)/{\text{N}}_{\text{S1}} \times 100\%$$

$${\text{Yield}}\left( \% \right) = {\text{N}}_{\text{P}} /{\text{Ns}}_{ 1} \times 100\%$$

N_S1_ is the initial moles of the substrate (mol), N_S2_ is the total moles of the substrate at equilibrium (mol), and N_P_ is the total moles of the product (mol) at equilibrium.

### Biosynthesis of cinnamyl alcohol from l-phenylalanine

Whole-cell catalysis of the transformation of l-phenylalanine to cinnamic acid was performed in 50 mL flasks that contained 3 mL of a reaction mixture consisting of 100 mM Tris–HCl buffer (pH 8.5), *E. coli* BLP3 (OD_600nm_ 15), and 40 mM l-phenylalanine. The reaction was performed at 55 °C with rotary shaking at 200 rpm for 2 h. Afterward, the mixture was heated to 100 °C and then centrifuged at 10,000*g* for 3 min. Subsequently, 102 mM glucose and a certain amount of cells (OD_600nm_ 50) were added to 1.5 mL of the supernatant and 1.5 mL of 200 mM PBS (consisting of 204 mM glucose and *E. coli* BLCS cells at OD_600nm_ 100) were mixed together. The second reaction was started after the addition of 1.2 mL of dibutyl phthalate and was carried out at 30 °C and 200 rpm for 6 h.

### Analytical methods

HPLC analysis was carried out on an Agilent 1260 Series instrument together with an Eclipse XDB-C18 column (250 mm × 4.6 mm, 5 μm). The concentrations of cinnamyl alcohol, cinnamic acid, cinnamaldehyde, and 3-phenylpropanol were monitored by measuring the absorbance at 254 nm. The mobile phase consisted of acetonitrile (A), methanol (B) and 25 mM potassium phosphate buffer (pH 2.5) (C),and an elution gradient elution was used: the ratio of the mobile phase was changed from 1:2:7 (A:B:C) to 2:1:7, and the flow rate was increased from 0.8 mL/min to 0.9 mL/min for 5 min. After maintaining the ratio for 10 min, the ratio was changed back to 1:2:7, and the flow rate was decreased to 0.8 mL/min for 1 min. These parameters were maintained for 3 min after which the flow rate was decreased to 0.4 mL/min for 3 min. The flow rate was then increased and increasing to 0.8 mL/min for 8 min, which was then maintained for 10 min. The column temperature was set to 40 °C.

### Statistical analysis

Statistical analyses were performed using SPSS 25.0. The differences in the corresponding values between the groups were tested by one-way analysis of variance (ANOVA). *p* < 0.05 was considered statistically significant.

## Results and discussion

### Biosynthesis of cinnamyl alcohol from cinnamic acid by *E. coli* BLCS in the monophasic aqueous system

**Fig. 1:**
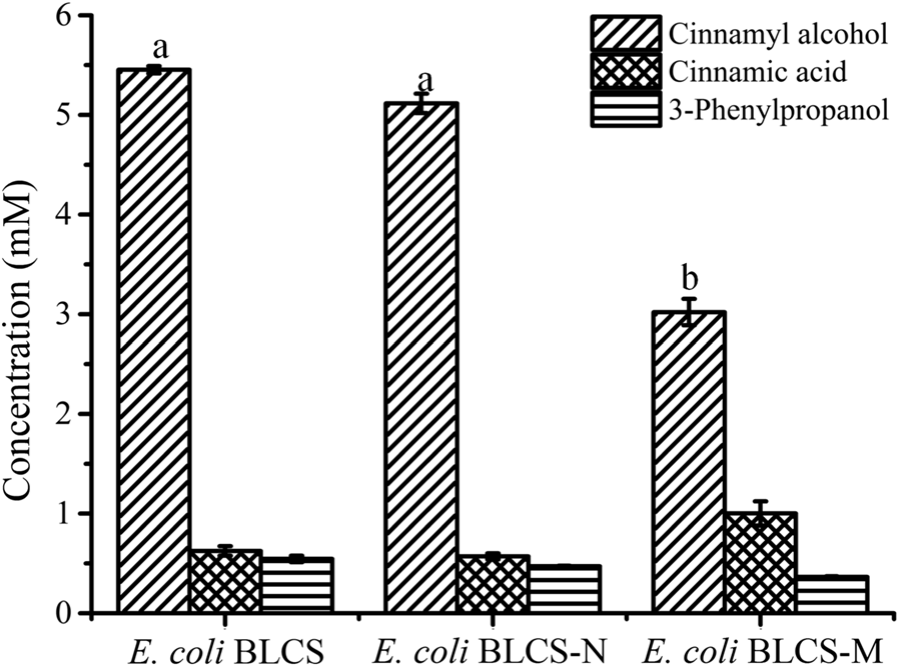
Comparison of the ability of different strains to synthesize cinnamyl alcohol. Conditions: 11 mM cinnamic acid, 55 mM glucose, different wet cells (OD_600 nm_ 50), 100 mM phosphate buffer (pH 7.5), 200 rpm, 42 °C, 3 mL final volume; the reaction lasted 4 h under the above conditions, with the different letters representing significant differences between the treatment means (*p *< 0.05)

Since the discovery of CAR in 1997, researchers have conducted many studies involving this enzyme [19]. CAR is a kind of enzyme with broad substrate specificity, and a variety of CARs exhibit cinnamic acid catalytic activity [20]. BsSFP can facilitate posttranslational modification of CAR and improve its activity [19, 21–25]. First, we screened MpCAR, NoCAR, and NiCAR, which have good cinnamic acid activity and coexpressed them in *E. coli* with BsSFP. To compare their ability to synthesize cinnamyl alcohol, the three strains were fed with 11.0 mM cinnamic acid. In addition, glucose was added as a cosubstrate to provide indispensable cofactors including NADH, NADPH and ATP. As shown in Fig. 1, the *E. coli* BLCS strain containing the pCDFDuet-NiCAR-BsSFP plasmid synthesized 5.5 mM cinnamyl alcohol, which was 44.6% and 6.2% higher than *E. coli* BLCS-M and *E. coli* BLCS-N, respectively. In addition, the concentrations of residual cinnamic acid, cinnamaldehyde and by-product 3-phenylpropanol were low. Thus, we focused on whole-cell biotransformation by *E. coli* BLCS.

### Limiting factors of the biosynthesis of cinnamyl alcohol by *E. coli* BLCS in the monophasic aqueous system

**Fig. 2:**
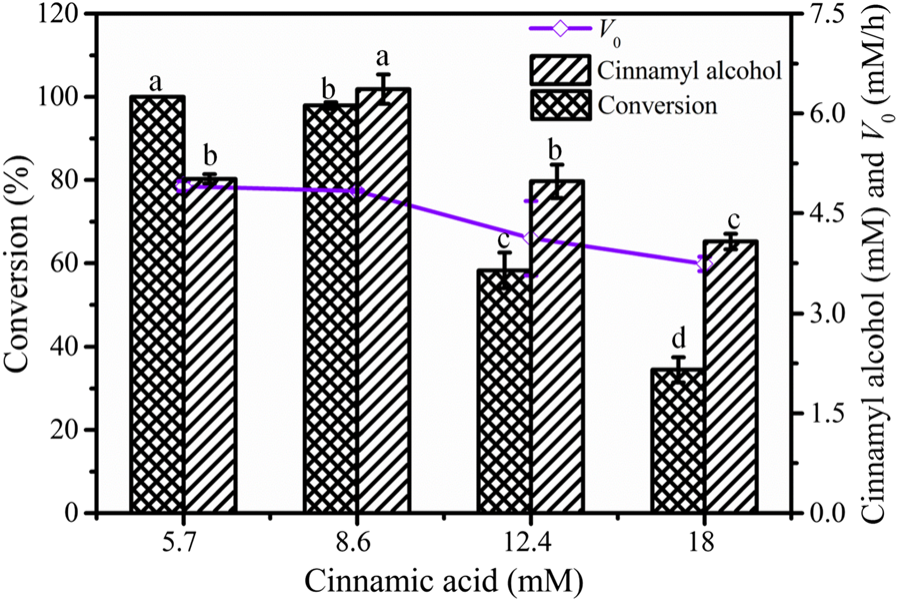
Effects of initial cinnamic acid concentration on whole-cell biotransformation. Conditions: 5.7–18 mM cinnamic acid, 55 mM glucose, wet cells (OD_600 nm_ 50), 100 mM phosphate buffer (pH 7.5), 200 rpm, 42 °C, 3 mL final volume; the reaction lasted 2 h under the above conditions, with the different letters representing significant differences between the treatment means (*p* < 0.05)

The effects of cinnamic acid on cinnamyl alcohol biosynthesis were studied. As shown in Fig. 2, the initial concentration of cinnamic acid exerted no significant effect on the conversion when cinnamic acid concentrations were lower than 8.6 mM. However, when the cinnamic acid concentration was greater than 8.6 mM, the conversion began to decrease obviously. In addition, *V*_0_ was 4.83 mM/h at the initial 8.6 mM cinnamic acid and it decreased by 22.6% when the substrate concentration reached 18 mM. The substrate was completely converted within 2 h, and the highest concentration of cinnamyl alcohol (6.37 mM) was obtained with a yield of 75.7% when the initial concentration of cinnamic acid was 8.6 mM. These results implied that substrate inhibition was obvious when the cinnamic acid concentration exceeded 8.6 mM, which was an obstacle to the production of cinnamyl alcohol.

The enzymes in this pathway may be temperature and pH dependent. By using a substrate concentration of 8.6 mM, we investigated the effects of temperature and pH on whole-cell catalysis. Additional file 1: Figure S3 shows that temperatures between 30 and 42 °C resulted in similar cinnamic acid conversions, and the optimum temperature for the synthesis of cinnamyl alcohol was 30 °C. As shown in Additional file 1: Figure S4, *E. coli* BLCS cells exhibited good catalytic performance within the pH range of 7.0–8.0. In addition, the conversions of cinnamic acid were 100%, and the yield of cinnamyl alcohol reached 7.37 mM. We further studied the effects of cell dosage and the molecular ratio of substrate to cosubstrate on the biosynthesis of cinnamyl alcohol to optimize catalytic conditions. As shown in Additional file 1: Figures S5, S6, the optimum cell dosage was OD_600nm_ 50, and the optimum molecular ratio of substrate to co-substrate was 1:6. Under the optimal conditions, the cinnamic acid was completely converted, and 7.51 mM cinnamyl alcohol accumulated in 2 h with a yield of 92.8%.

**Fig. 3:**
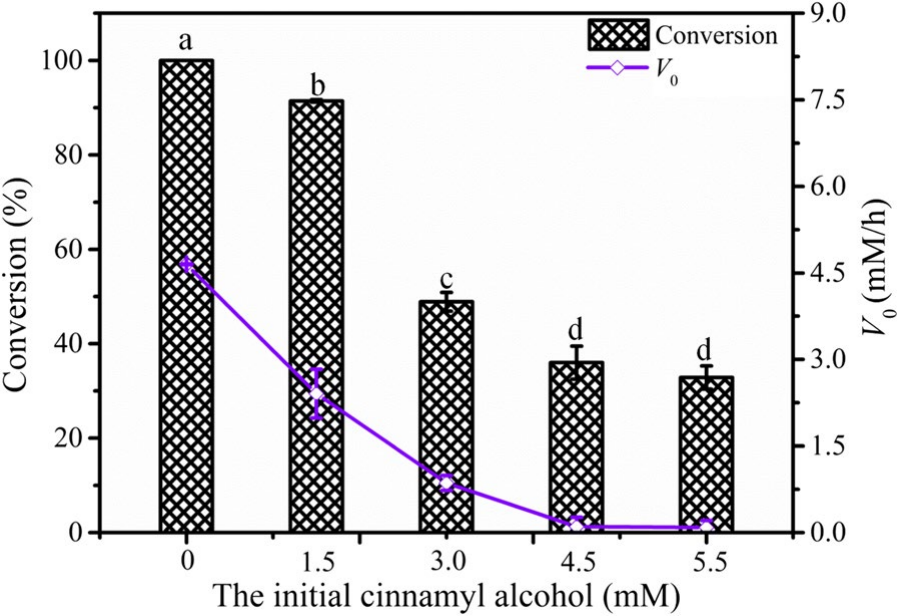
Effects of initial cinnamyl alcohol concentration on whole-cell biotransformation. Conditions: 0–5.5 mM cinnamyl alcohol, 8.5 mM cinnamic acid, 51 mM glucose, wet cells (OD_600nm_ 50), 100 mM phosphate buffer (pH 7.5), 200 rpm, 30 °C, 3 mL final volume; the reaction lasted 2 h under the above conditions, with different the letters representing significant differences between the treatment means (*p* < 0.05)

It is possible that the presence of product could have a negative effect on the conversion or yield. Thus, the effects of the product on the biocatalytic cascade were also analyzed by adding cinnamyl alcohol at the beginning of the reaction. As shown in Fig. 3, the initial cinnamyl alcohol concentrations obviously affected the conversion of cinnamic acid. When the initial concentration of cinnamyl alcohol was 5.5 mM, the conversion of cinnamic acid considerably decreased to 32.8%. In addition, the *V*_0_ was 4.65 mM/h without the addition of cinnamyl alcohol and it decreased substantially with the increase in the initial concentration of cinnamyl alcohol. When the initial concentration of cinnamyl alcohol reached 5.5 mM, the *V*_0_ decreased by 90.2%. This phenomenon indicated that cinnamyl alcohol results in strong feedback inhibition, which was even more severe than substrate inhibition. At present, there are no relevant reports showing that the product inhibition of cinnamyl alcohol is the main bottleneck for its biosynthesis.

### Selection of organic solvent for cinnamyl alcohol biosynthesis

Several approaches have been used to address product or substrate inhibition, such as adding organic solvents to reduce their concentration in the aqueous phase or altering the structure of enzymes using site-directed mutagenesis [26–29]. A biphasic system was chosen to address the problem in this work.

We investigated the distribution of cinnamic acid and its downstream products in six organic solvents. As shown in Table 1, cinnamic acid exhibited extensive hydrophilicity in different organic solvents. Most of the cinnamic acid was distributed in the aqueous phase, which is beneficial for microbes to acquiring substrates. In addition, a portion of the cinnamic acid was dissolved in the organic phase and it would gradually enter the aqueous phase as its content in the aqueous phase decreased during biotransformation. Thus, this phenomenon may be conductive to relieving substrate inhibition. As the reduction product of cinnamic acid, cinnamaldehyde is cytotoxic to microorganisms [2]. It showed good lipophilicity in all six organic solvents, and dibutyl phthalate (with the highest log*P* value) demonstrated the strongest solubility for cinnamaldehyde. It was proposed that using dibutyl phthalate as an organic solvent may be beneficial for removing cinnamaldehyde in the water phase and for reducing its toxicity to microorganisms. However, whether this removal leads to a reduction in the end product still requires further experiments. Compared with cinnamic acid and cinnamaldehyde, the terminal product cinnamyl alcohol exhibited moderate lipophilicity in the organic solvents. With the exceptions of *n*-hexane and *n*-octane, the other four organic solvents, especially *n*-octanol and *n*-hexanol, could effectively extract cinnamyl alcohol from the aqueous phase, suggesting that those solvents could be used to relieve the inhibition of cinnamyl alcohol for bioconversion.

**Table 1:** Distribution coefficients of cinnamic acid and its derivatives in different biphasic systems

Organic solvent	Log*P*^a^	Log*P* cinnamic acid	Log*P* cinnamyl alcohol	Log*P* cinnamaldehyde
Dibutyl phthalate	5.4	− 0.65	1.55	3.19
*n*-Octane	4.5	− 2.16	0.22	1.62
*n*-Hexane	3.5	− 4.53	− 0.42	1.29
*n*-Octanol	2.9	− 0.50	1.73	1.90
Ethyl acetate	1.7	− 0.46	1.55	1.98
*n*-Hexanol	1.4	− 0.36	1.98	2.73

^a^Data from Laane et al. [30]

**Fig. 4:**
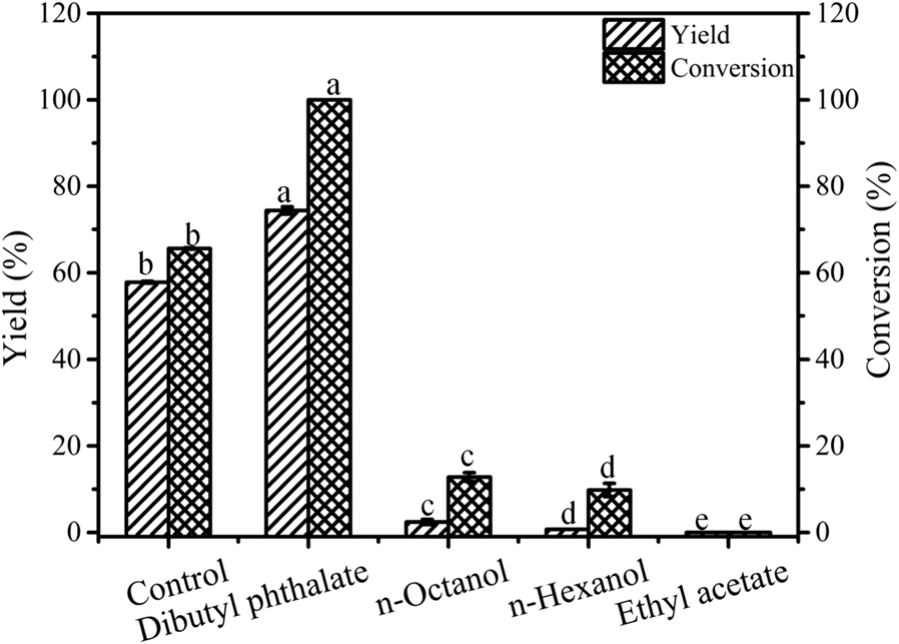
Effects of various organic solvents on bioconversion by whole-cell catalysis in biphasic system. Conditions: 3 mL of 100 mM phosphate buffer (pH 7.5) consisting of 17.4 mM cinnamic acid, 102 mM glucose, wet cells (OD_600nm_ 50) and 3 mL of various organic solvents were mixed together at 200 rpm and 30 °C for 6 h under the above conditions, with different letters representing significant the differences between the treatment means (*p* < 0.05)

With respect to biphasic biotransformation, organic solvents not only influence the distribution of compounds, but also are toxic to microorganisms and affect the activity of cells [31]. Thus, the four organic solvents and an equal volume of water phase (consisting of 17.4 mM cinnamic acid) were mixed together to construct a biphasic system. The effects of the different organic solvents on the bioconversion were shown in Fig. 4. A yield of 57.8% and conversion of 65.6% were obtained using the monophasic system. With the use of a biphasic system, the other three organic solvents did not work well, indicating that they showed greater molecular toxicity to microbial cells and are not conducive to biotransformation. In the dibutyl phthalate-aqueous system, cinnamic acid was totally converted with a cinnamyl alcohol yield of 74.7%. Moreover, only a small amount of cinnamyl alcohol was present in the aqueous phase (Additional file 1: Table S2). As expected, dibutyl phthalate maintained the cell activity at a relatively good level, and the dibutyl phthalate-aqueous system could not only promote biotransformation by removing product inhibition, but also realize the simultaneous synthesis and separation of cinnamyl alcohol. Hence, dibutyl phthalate was selected as a suitable organic phase of the biphasic system for the biotransformation of cinnamic acid to cinnamyl alcohol.

### Effects of the phase ratio on bioconversion in the biphasic system

**Fig. 5:**
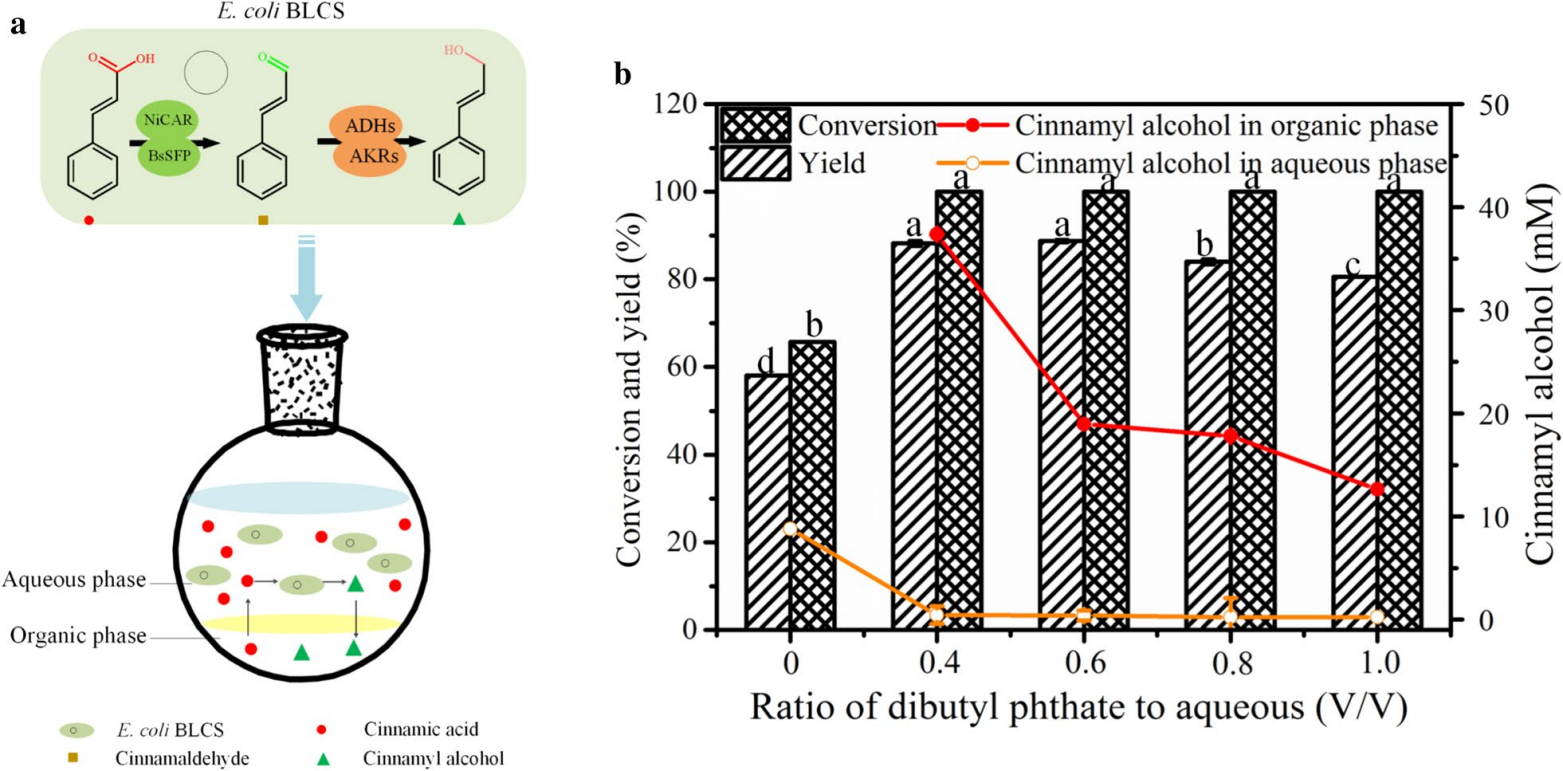
Schematic of the whole-cell catalysis of cinnamic acid to cinnamyl alcohol by *E. coli* BLCS in a biphasic system (**a**). Effects of volumetric ratio of the organic phase to the aqueous phase on the biosynthesis of cinnamyl alcohol (**b**). Conditions: 3 mL of 100 mM phosphate buffer (pH 7.5) consisting of 17.4 mM cinnamic acid, 102 mM glucose, wet cells (OD_600nm_ 50) and 0–3 mL of dibutyl phthalate were mixed together at 200 rpm, 30 °C; the reaction lasted 6 h under the above conditions, with the different letters representing significant differences between the treatment means (*p *< 0.05)

In an organic-aqueous biphasic system, the volumetric phase ratio influences both the interfacial areas and cell viability [31–34]. These influences may affect microbial glucose metabolism, which indirectly affects the ability of cells to supply cofactors. Thus, the effects of the ratio of organic to aqueous phases on bioconversion were evaluated. As shown in Fig. 5b, once the organic phase was used, 17.4 mM cinnamic acid could be completely transformed in 6 h. The cinnamyl alcohol yield decreased slightly with the increase in the ratio of organic to aqueous phase (v/v), and higher yields were observed at the ratios of 0.4 and 0.6. Moreover, the ratio had a significant effect on the cinnamyl alcohol concentration in the organic phase, and it reached the maximum at the ratio of 0.4. As a result, the optimal ratio of the dibutyl phthalate phase to the aqueous phase was considered to be 0.4. Under these conditions, the yield was 88.2%, and cinnamyl alcohol in the organic phase reached 37.4 mM (corresponding to 5.02 g/L). The highest reported level of cinnamyl alcohol biosynthesis was approximately 4.8 mM [17] and it reached 10.99 mM (based on the total system) in this study, which was 143.1% higher than previously reported values. Moreover, the system synchronized the reaction, separation and concentration (Fig. 5a); hence, we could directly obtain the product from the organic phase at the level of grams per liter, which was conducive to product purification. Therefore, subsequent experiments were performed in the dibutyl phthalate-aqueous system with a volume ratio of 0.4.

### Effects of substrate concentrations on the production of cinnamyl alcohol in the biphasic system

The effects of substrate concentration on bioconversion were further evaluated in the biphasic system. As shown in Table 2, increasing cinnamic acid concentration to 24.7 mM still resulted in complete conversion (100%). However, the yield of cinnamyl alcohol decreased obviously and the amount of cinnamaldehyde in the organic phase increased significantly with increasing substrate concentration. In this work, the biosynthesis of cinnamyl alcohol from cinnamic acid involved two steps: cinnamaldehyde production by the recombinant NiCAR and cinnamaldehyde bioreduction by endogenous ADHs and AKRs of *E. coli*. Thus the imbalance of activities between the recombinant NiCAR and endogenous reductases would result in the accumulation of cinnamaldehyde, but a low level of cinnamaldehyde in the monophasic system seemed to prevent this possibility. When the biphasic system is used, taking account of dibutyl phthalate’s good ability to extraction cinnamaldehyde, it can be reasonably expected that the cinnamaldehyde produced after the 1st step has two outlets: one is to synthesize corresponding alcohol continually in microbes via the 2nd step, and the other involves being released from the cells and entering into the organic phase. There is a competitive relation between the above two pathways. Once cinnamaldehyde entered into the organic phase, the 2nd reaction did not occur because the cells could not effectively come into contact with cinnamaldehyde in dibutyl phthalate. In addition, cinnamaldehyde is cytotoxic and the intracellular accumulation of cinnamaldehyde has a negative effect on cell vitality, which in turn affects the biosynthesis of cinnamyl alcohol. Therefore, as the substrate concentration increased, an increasing amount of cinnamaldehyde was produced, which contributed to the distribution of cinnamaldehyde in the organic phase. It was supposed that this problem might be solved by overexpression of other ADH and AKR with higher activity.

**Table 2:** Effects of initial substrate concentration in the aqueous phase on the biosynthesis of cinnamyl alcohol in the biphasic system

The initial substrate in the aqueous phase (mM)	Organic phase (mM)		Aqueous phase (mM)		Yield (%)	Conversion (%)
	Cinnamyl alcohol	Cinnamaldehyde	Cinnamyl alcohol	Cinnamaldehyde		
17.4	37.36 ± 0.24	4.34 ± 0.19	0.41 ± 0.06	0.13 ± 0	88.2 ± 0.56	100 ± 0.05
24.7	34.13 ± 0	22.84 ± 0	0.52 ± 0	0.21 ± 0	59.0 ± 0	100 ± 0.04
32.0	27.44 ± 0	31.40 ± 0	0.27 ± 0	0.22 ± 0	45.5 ± 0	77.2 ± 0.03

Conditions: 3 mL of 100 mM phosphate buffer (pH 7.5) consisting of 17.432 mM cinnamic acid, 102 mM glucose, wet cells (OD_600nm_ 50) and 1.2 mL of dibutyl phthalate, 200 rpm, 30 °C; the reaction lasted 6 h under the above conditions

### Biosynthesis of cinnamyl alcohol from l-phenylalanine

**Fig. 6:**
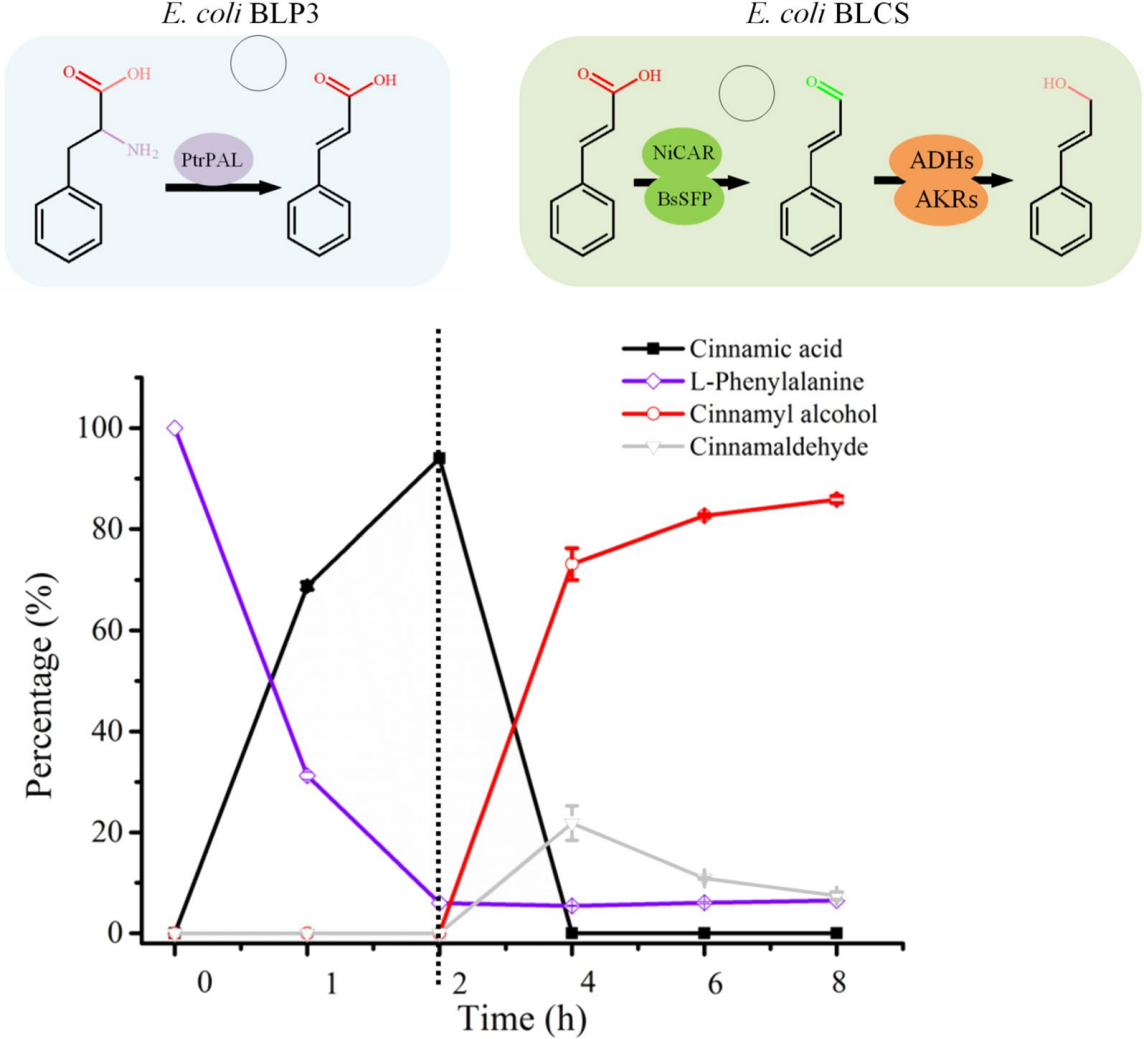
Time-course profiles of cinnamyl alcohol biosynthesis from l-phenylalanine. Conditions: 3 mL of 100 mM phosphate buffer (pH 8.5) consisting of 40 mM l-phenylalanine, *E. coli* BLP3 (OD_600nm_ 15), 200 rpm, 30 °C; the reaction lasted 2 h under the above conditions to synthesize cinnamic acid. The reaction mixture was spun down, after which the 1.5 mL of the supernatant was supplemented with 102 mM glucose and *E. coli* BLCS (OD_600 nm_ 50) until the volume of the aqueous phase reached 3 mL. The second reaction was started after the addition of 1.2 mL of dibutyl phthalate and was carried out at 30 °C and 200 rpm for 6 h

In terms of industrialization, the synthesis of cinnamyl alcohol from inexpensive substrates such as glucose is the direction headed. Considering that l-phenylalanine has been successfully produced from glucose by fermentation with high yields [35–38], we studied the biosynthesis of cinnamyl alcohol from l-phenylalanine. Previously, we found that recombinant *E. coli* BLP3 overexpressing PAL3 from *P. trichocarpa* (PtrPAL3) could synthesize cinnamic acid from l-phenylalanine efficiently by whole-cell biotransformation [39]. Therefore, we chose this strain to synthesize cinnamic acid, which was coupled with the synthesis of cinnamyl alcohol in the biphasic system. The coupled reaction was performed in a sequential manner. A full time course experiment of the two biocatalytic systems was performed, the results of which are shown in Fig. 6. l-Phenylalanine (40 mM) was converted to cinnamic acid in 2 h by *E. coli* BLP3, with a conversion of 87.2%. The supernatant (1.5 mL) was added to the aqueous phase (the volume increased to 3 mL) to conduct the subsequent reaction by *E. coli* BLCS in the biphasic system for 6 h. Finally, 0.049 mmol cinnamyl alcohol was synthesized from 0.06 mmol l-phenylalanine with a yield of 81.7% after 8 h. Moreover, the concentration of cinnamyl alcohol in the organic phase reached 38.23 mM with a high purity of 92.9% (Additional file 1: Table S3). Klumbys et al. used a three-step cascade to convert l-phenylalanine to cinnamyl alcohol (4.3 mM) in 27.5 h [13]. In contrast, the coupling process established in this study had higher cinnamyl alcohol synthesis efficiency, suggesting that the method may be applicable to the de novo biosynthesis of cinnamyl alcohol.

## Conclusion

Herein, we focused on the key module of the cinnamyl alcohol biosynthesis pathway, reducing cinnamic acid to cinnamyl alcohol. An efficient biotransformation approach was successfully established using recombinant *E. coli* BLCS coexpressing *NiCAR* and *BsSFP* in this work. A biphasic system was indicated to be an effective strategy for improving the catalytic performance of microbial cells. The use of dibutyl phthalate successfully removed cinnamyl alcohol from the aqueous phase, thus avoiding severe product inhibition and realizing the concentration of cinnamyl alcohol simultaneously. Up to 17.4 mM cinnamic acid was reduced to the desired product, with a yield of 88.2% in 6 h and the synthesized cinnamyl alcohol was concentrated to 37.36 mM in the organic phase. Furthermore, the integration of this process with cinnamic acid production demonstrated a robust performance for cinnamyl alcohol production from l-phenylalanine. These findings open up possibilities for the practical biosynthesis of natural cinnamyl alcohol at an industrial scale.

## Supplementary information

**Additional file 1: Table S1.** Strains and plasmids used in this study. **Table S2.** Effects of various organic solvents on the concentration of cinnamyl alcohol in the biphasic system. **Table S3.** The proportion of organic phase components in time-course profiles of cinnamyl alcohol biosynthesis from l-phenylalanine. **Figure S1.** Biosynthetic pathways from l-phenylalanine to cinnamyl alcohol. **Figure S2.** HPLC analysis of sample of *E. coli* BLCS transformation cinnamic acid after 2 h. (a) Cinnamic acid and its derivatives standard samples; (b) sample for HPLC analysis was taken at the time of 2 h biotransformation. **Figure S3.** Effects of temperature on whole-cell biotransformation. **Figure S4.** Effects of pH on whole-cell biotransformation. **Figure S5.** Effects of cell dosage on whole-cell biotransformation. **Figure S6.** Effects of the ratio of cinnamic acid to glucose on whole-cell biotransformation.

## Data Availability

All data generated or analyzed during this study are included in this manuscript.
