# Supplementary material for: Efficient biosynthesis of cinnamyl alcohol by engineered Escherichia coli overexpressing carboxylic acid reductase in a biphasic system
Source: Microb Cell Fact. 2020 Aug 12;19:163. doi: 10.1186/s12934-020-01419-9 (PMC7424670; doi:10.1186/s12934-020-01419-9)
Supplement: Supplementary file 1 — Additional file 1: Table S1. Strains and plasmids used in this study. Table S2. Effects of various organic solvents on the concentration of cinnamyl alcohol in the biphasic system. Table S3. The proportion of organic phase components in time-course profiles of cinnamyl alcohol biosynthesis from l-phenylalanine. Figure S1. Biosynthetic pathways from l-phenylalanine to cinnamyl alcohol. Figure S2. HPLC analysis of sample of E. coli BLCS transformation cinnamic acid after 2 h. (a) Cinnamic acid and its derivatives standard samples; (b) sample for HPLC analysis was taken at the time of 2 h biotransformation. Figure S3. Effects of temperature on whole-cell biotransformation. Figure S4. Effects of pH on whole-cell biotransformation. Figure S5. Effects of cell dosage on whole-cell biotransformation. Figure S6. Effects of the ratio of cinnamic acid to glucose on whole-cell biotransformation. [file 12934_2020_1419_MOESM1_ESM.doc]

Additional Information for

Efficient biosynthesis of cinnamyl alcohol by engineered *Escherichia coli* overexpressing carboxylic acid reductase in a biphasic system

*Chen Zhang^1, 2^*^#^*, Qian Xu^1^*^#^*, Hongliang Hou^1^, Jiawei Wu^1^, Zhaojuan Zheng^1, 3^, Jia Ouyang^1, 2 *^*

^1^ Jiangsu Co-Innovation Center of Efficient Processing and Utilization of Forest Resources, College of Chemical Engineering, Nanjing Forestry University, Nanjing 210037, People’s Republic of China

^2^ Key Laboratory of Forestry Genetics & Biotechnology (Nanjing Forestry University), Ministry of Education, Nanjing 210037, People’s Republic of China

^3^ Jiangsu Province Key Laboratory of Green Biomass-based Fuels and Chemicals, Nanjing 210037, People’s Republic of China

^#^ These authors contributed equally to this work.

* Corresponding Author Address: College of Chemical Engineering, Nanjing Forestry University, Nanjing 210037, People’s Republic of China, Tel.: 86-025-85427129, Fax: 86-025-85427587, E-mail: [hgouyj@njfu.edu.cn](mailto:hgouyj@njfu.edu.cn).

**Table S1**. Strains and plasmids used in this study.

| Strains, plasmids, and primers | Description | Source |
| --- | --- | --- |
| **Strains** |  |  |
| *E. coli* BL21 (DE3) | F^−^ ompT hsdSB (rB^−^ mB^−^) gal (λ cI857 ind1 sam7 nin5 lacI lacUV5-T7 gene1), dcm (DE3) | Transgen |
| *E. coli* BLP3 | BL21(DE3) carrying pETDuet-PtrPAL3 | Laboratory preservation |
| *E. coli* BLCS | BL21(DE3) carrying pCDFDuet-NiCAR-BsSFP | This study |
| *E. coli* BLCS-N | BL21(DE3) carrying pCDFDuet-NoCAR-BsSFP | This study |
| *E. coli* BLCS-M | BL21(DE3) carrying pCDFDuet-MpCAR-BsSFP | This study |
| **Plasmids** |  |  |
| pCDFDuet-1 | Double T7 promoter, CDF ori, Sm^r^ | Novagen |
| pETDuet-1 | Double T7 promoter, pBR322 ori, Amp^r^ | Novagen |
| pCDFDuet-NiCAR | pCDFDuet-1 carrying *NiCAR* | This study |
| pCDFDuet-NiCAR-BsSFP | pCDFDuet-1 carrying *NiCAR* and *BsSFP* | This study |
| pCDFDuet-NoCAR | pCDFDuet-1 carrying *NoCAR* | This study |
| pCDFDuet-NoCAR-BsSFP | pCDFDuet-1 carrying *NoCAR* and *BsSFP* | This study |
| pCDFDuet-MpCAR | pCDFDuet-1 carrying *MpCAR* | This study |
| pCDFDuet-MpCAR-BsSFP | pCDFDuet-1 carrying *MpCAR* and *BsSFP* | This study |

**Table S2.** Effects of various organic solvents on the concentration of cinnamyl alcohol in the biphasic system.

| Organic solvents | Organic phase (mM) | Aqueous phase (mM) |
| --- | --- | --- |
| Dibutyl phthalate | 12.70±0.11 | 0.62±0.05 |
| *n*-Octanol | 0.43±0.10 | 0 |
| *n*-Hexanol | 0.13±0.00 | 0 |
| Ethyl acetate | 0 | 0 |

**Table S3.** The proportion of organic phase components in time-course profiles of cinnamyl alcohol biosynthesis from L-phenylalanine.

| Time（h） | Cinnamyl alcohol (%) | Cinnamaldehyde (%) | 3-Phenylpropanol (%) |
| --- | --- | --- | --- |
| 2 | 76.38±3.52 | 22.74±3.53 | 0.42±0.01 |
| 4 | 87.67±0.05 | 11.16±0.04 | 0.52±0.01 |
| 6 | 92.02±0.80 | 7.19±0.81 | 0.34±0 |


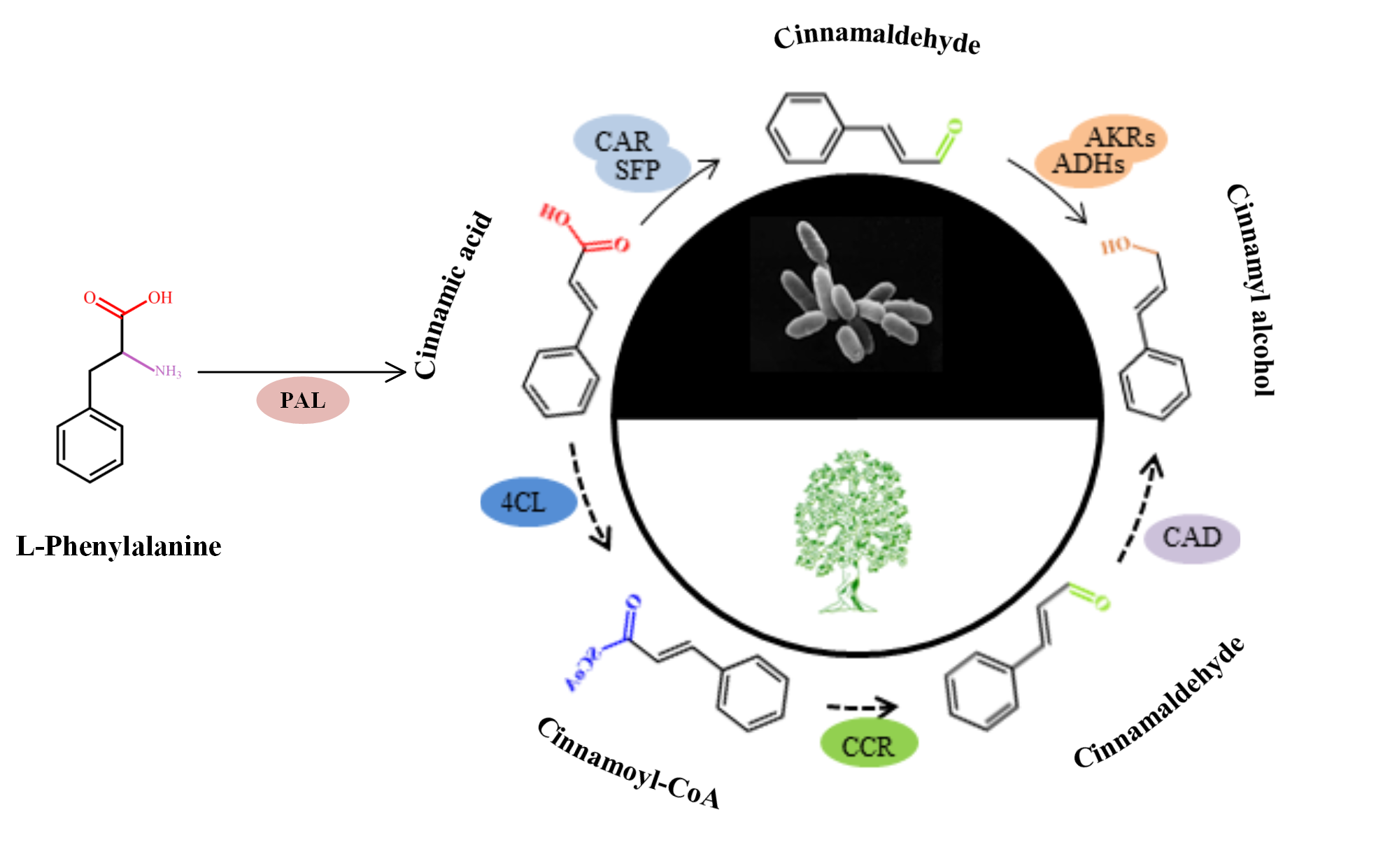


**Figure S1** Biosynthetic pathways from L-phenylalanine to cinnamyl alcohol. PAL: phenylalanine ammonia lyase; 4CL: 4-coumarate: CoA ligase; CCR: cinnamoyl-CoA reductase; CAD: cinnamyl alcohol dehydrogenase; CAR: carboxylic acid reductase; SFP: phosphopantetheine transferase; ADH: alcohol dehydrogenase; AKR: aldo-keto reductase.


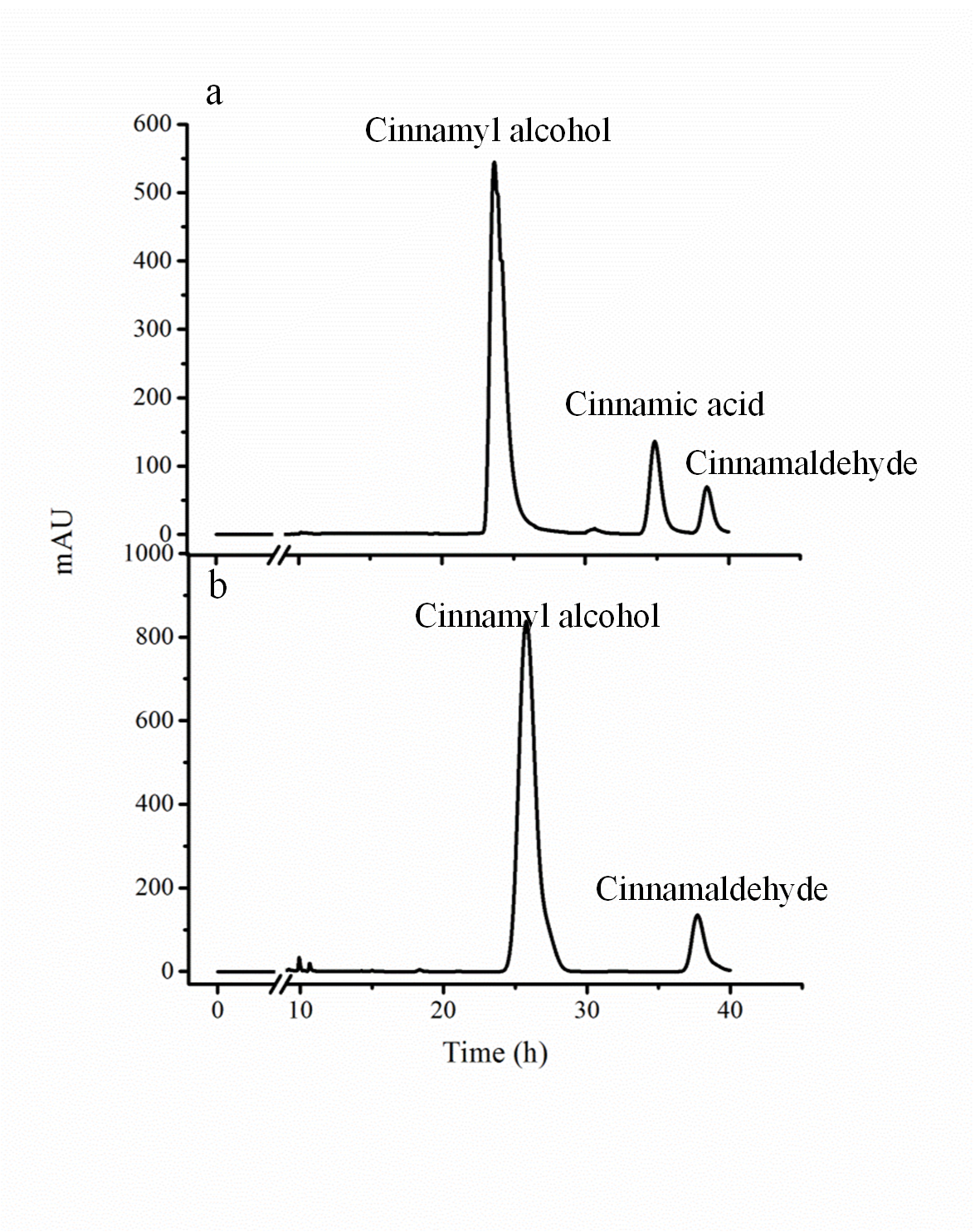


**Figure S2** HPLC analysis of sample of *E. coli* BLCS transformation cinnamic acid after 2 h. (a) Cinnamic acid and its derivatives standard samples; (b) sample for HPLC analysis was taken at the time of 2 h biotransformation.


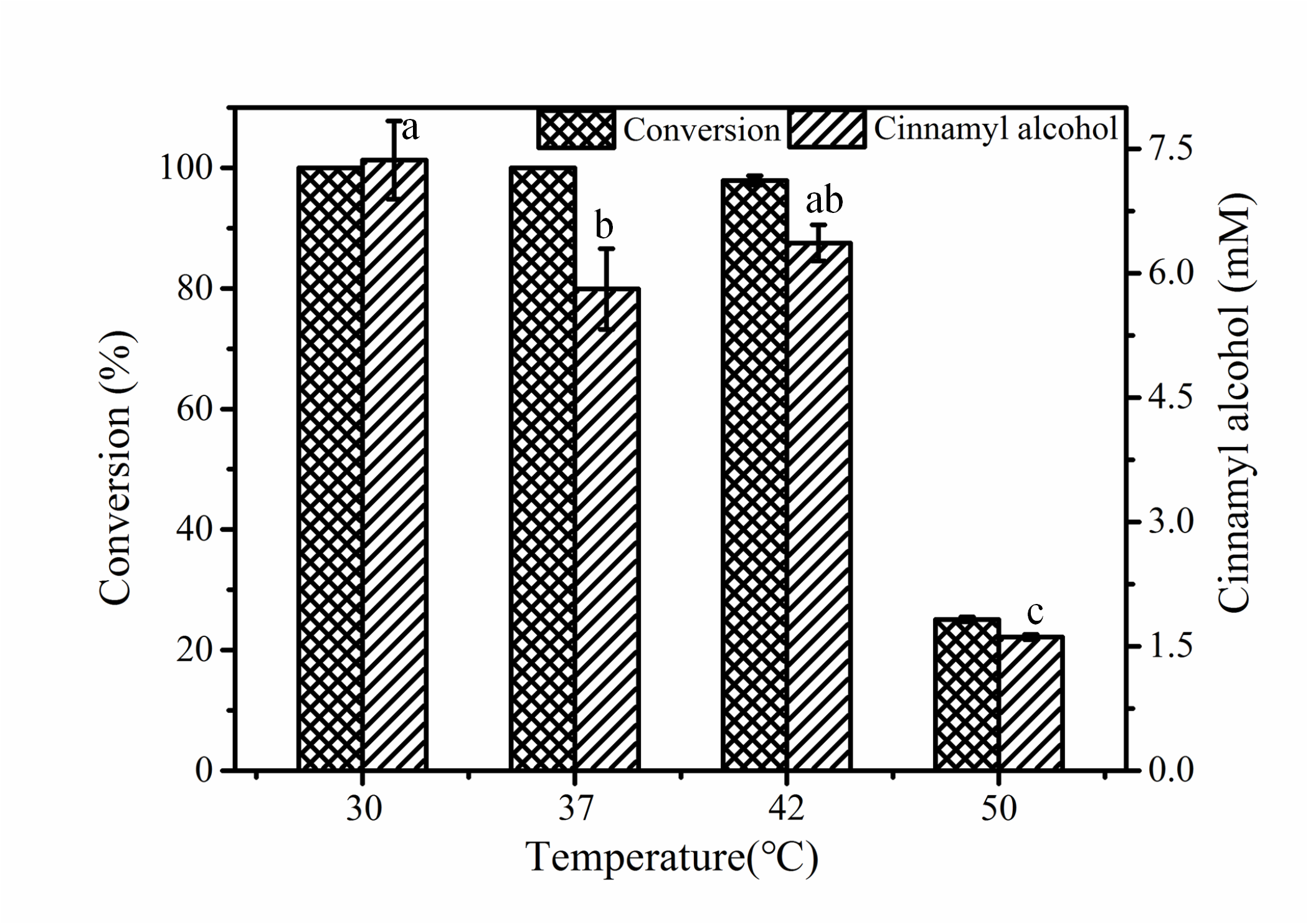


**Figure S3** Effects of temperature on whole-cell biotransformation. Conditions: 8.5 mM cinnamic acid, 55 mM glucose, OD_600 nm_ 50, 100 mM phosphate buffer (pH 7.5), 200 rpm, 30-50 ℃, 3 mL final volume; reaction lasted 2 h under the above conditions, with different letters representing significant differences between the treatment means (*p* < 0.05).


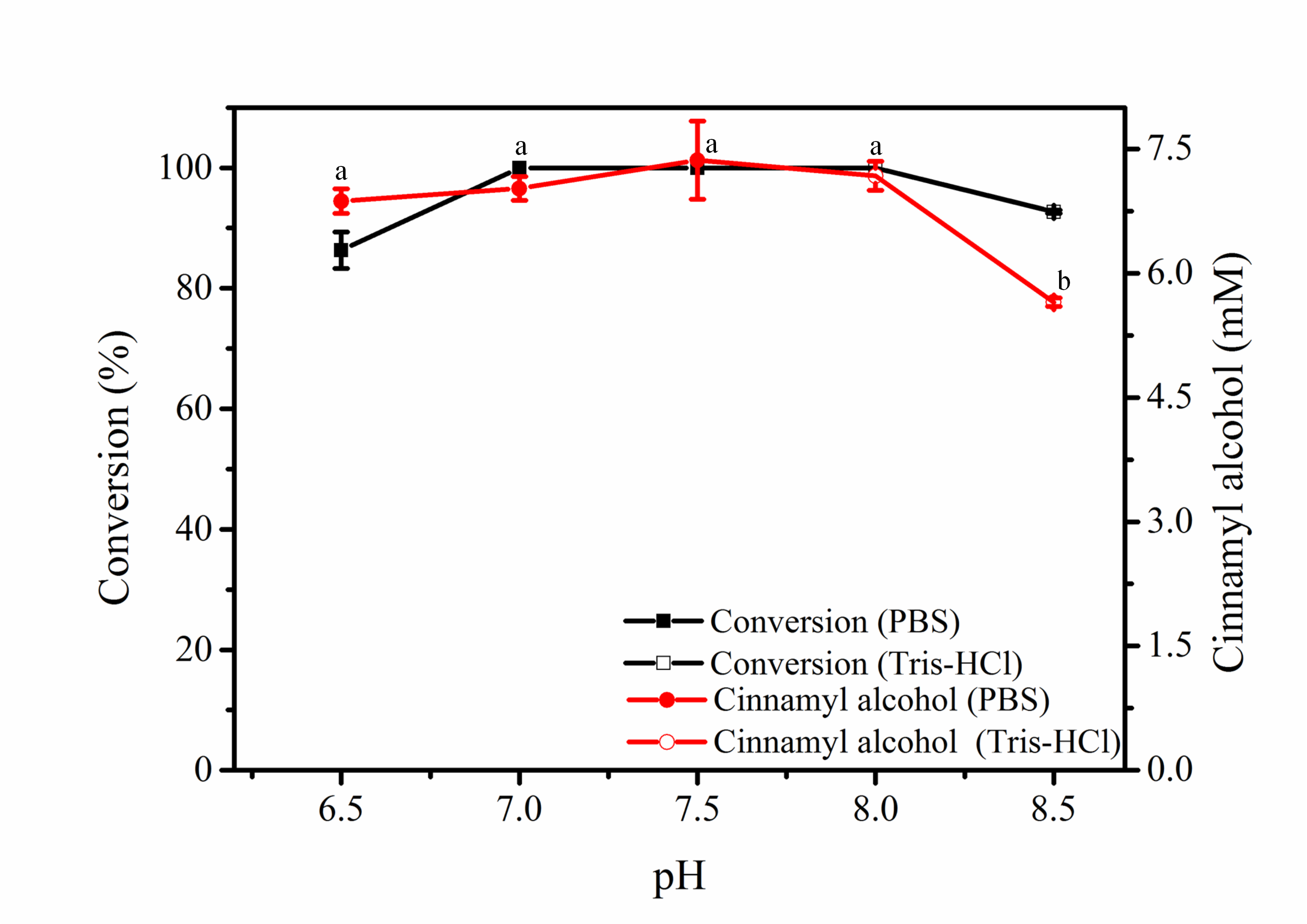


**Figure S4** Effects of pH on whole-cell biotransformation. Conditions: 8.5 mM cinnamic acid, 55 mM glucose, OD_600 nm_ 50, 100 mM phosphate buffer (pH 6.5-7.5) or Tris-HCl (pH 7.5-8.5) , 200 rpm, 30 ℃, 3 mL final volume; reaction lasted 2 h under the above conditions, with different letters representing significant differences between the treatment means (*p* < 0.05).


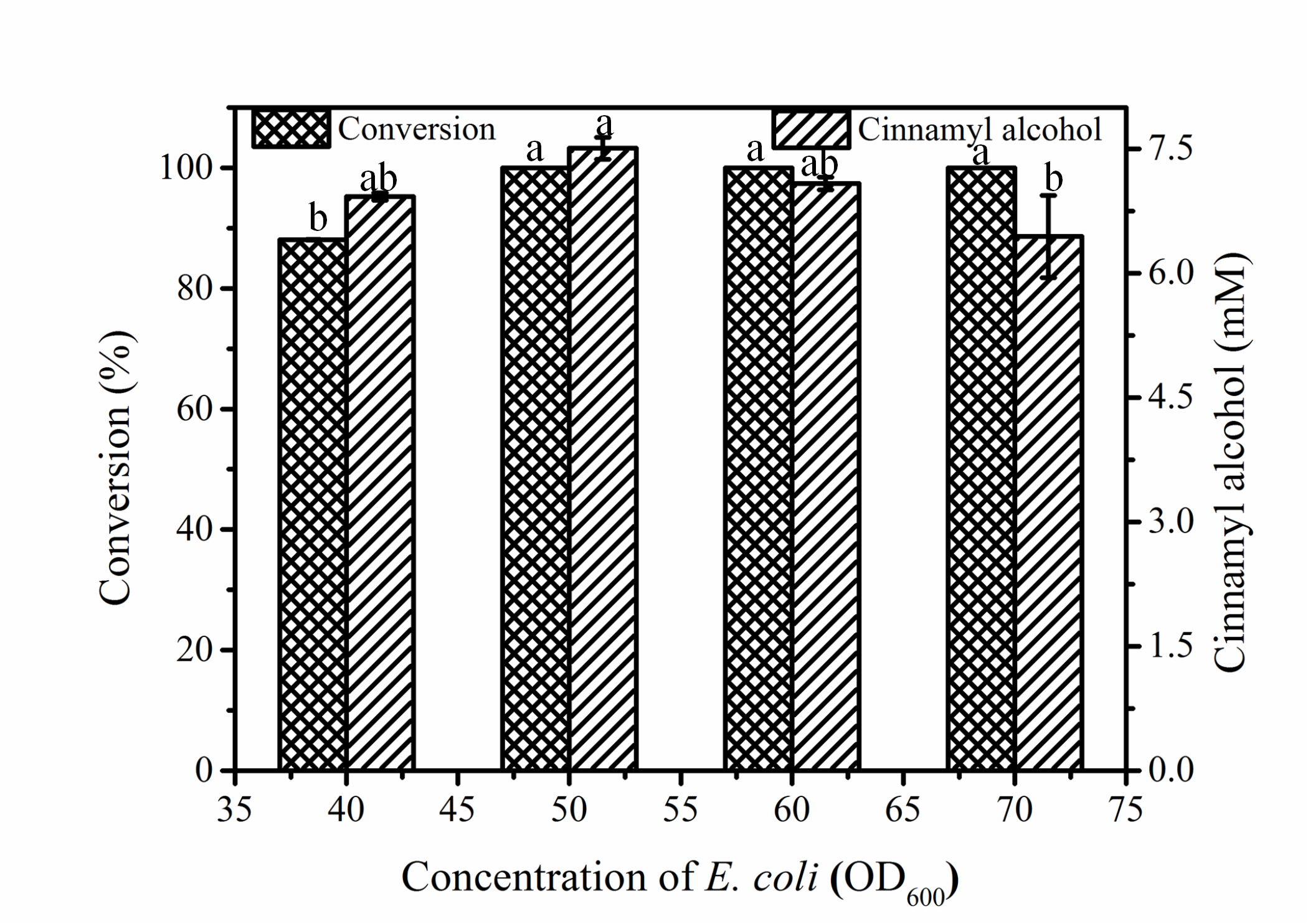


**Figure S5** Effects of cell dosage on whole-cell biotransformation. Conditions: 8.5 mM cinnamic acid, 55 mM glucose, OD_600 nm_ 40-70, 100 mM phosphate buffer (pH 7.5), 200 rpm, 30 ℃, 3 mL final volume; reaction lasted 2 h under the above conditions, with different letters representing significant differences between the treatment means (*p* < 0.05).


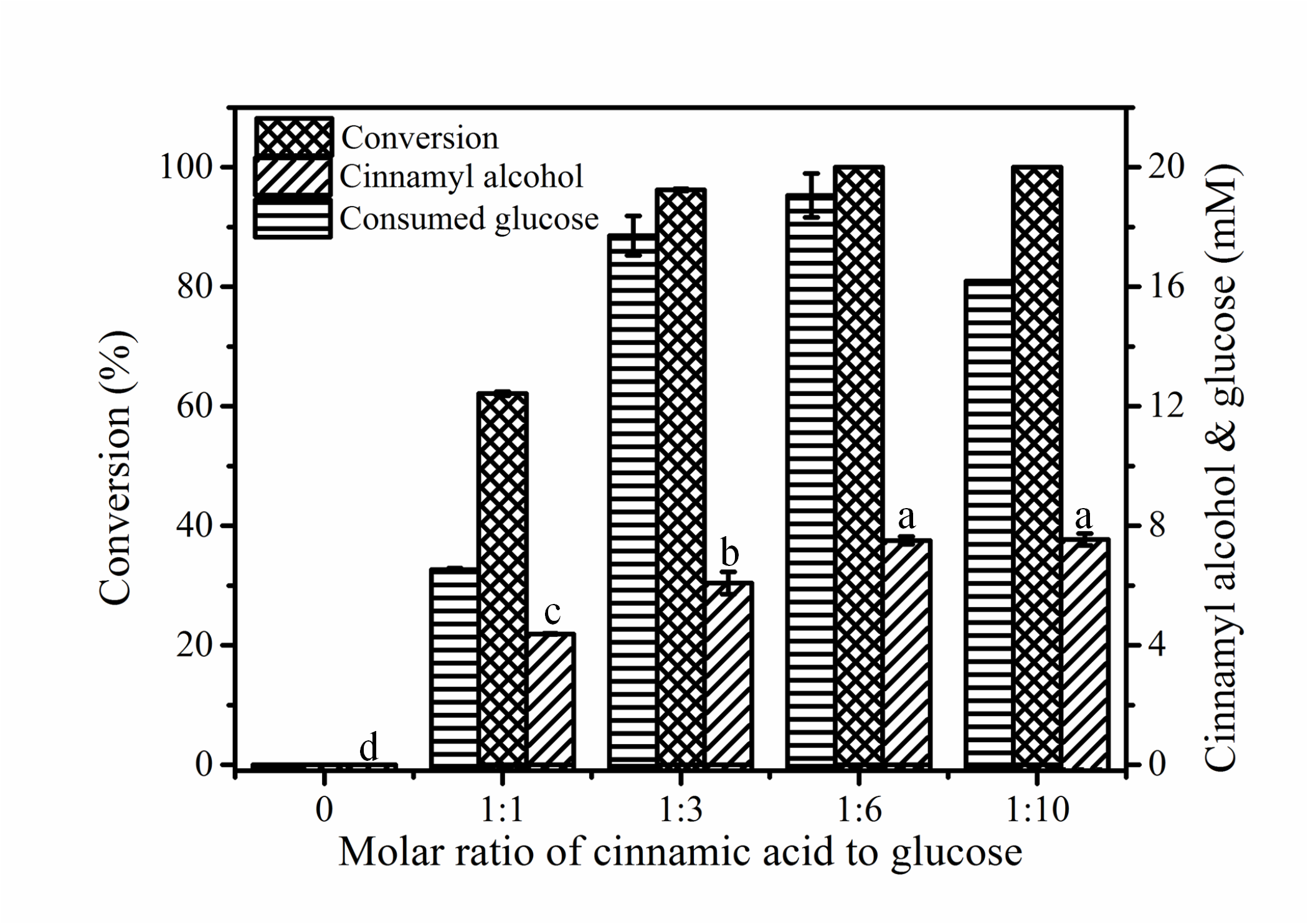


**Figure S6** Effects of the ratio of cinnamic acid to glucose on whole-cell biotransformation. Conditions: 8.5 mM cinnamic acid, ratio 0-1:6, OD_600 nm_ 40-70, 100 mM phosphate buffer (pH 7.5), 200 rpm, 30 ℃, 3 mL final volume; reaction lasted 2 h under the above conditions, with different letters representing significant differences between the treatment means (*p* < 0.05).
